# Seroprevalence Study of Hepatitis C and Hepatitis B Virus among Hospitalized Intravenous Drug Users in Ahvaz, Iran (2002-2006)

**Published:** 2010-06-01

**Authors:** Seyed Mohammad Alavi, Fatemeh Behdad

**Affiliations:** 1 Infectious and Tropical Disease Research Center Ahvaz Jundishapur University of Medical Sciences, Ahvaz, Iran; 2 Infectious Disease Division, Razi Hospital, Ahvaz Jundishapur University of Medical Sciences, Ahvaz, Iran

**Keywords:** Intravenous Drug Users, Viral Hepatitis, HBV, HCV

## Abstract

**Background and Aims:**

Viral hepatitis is a serious complication among intravenous drug users (IDUs). The objectives of this study were to determine the seroprevalence of hepatitis B and C viruses (HBV and HCV), and associated risk factors among IDUs at a teaching hospital in Ahvaz, southwest Iran.

**Methods:**

Medical records of 333 IDUs hospitalized from 2002 to 2006 at Razi Hospital, which is affiliated to Ahvaz Jundishapur University of Medical Sciences, were reviewed. Cases meeting the criteria for a diagnosis of viral hepatitis infection were included in this study. Patients’ characteristics, clinical and laboratory findings were extracted. Data of cases with hepatitis virus infection (HVI), called the HVI group and without HVI, called the NHVI group, were compared, using the chi-square test for qualitative variables and the t-test for quantitative variables. Differences with a P < 0.05 were considered significant.

**Results:**

Out of a total of 333 IDUs, 115 (34.5%), mostly male, with a mean age of 24.8±6.2 had HVI. More than 65% had a history of imprisonment. The mean duration of IDU was 4.5±1.6 years for the HVI group and 1.8±0.4 years for the NHVI group (P < 0.05). 85% of the HVI group and 45% of the NHVI group shared injection equipment (P < 0.05). 103 patients (30.9%) had HCV and 12 (3.6%) had HBV infection. There was a significant difference in age, duration of drug abuse, time spent in prison, sharing injection equipment, history of surgery, blood transfusion, packs of cigarettes per year and human immunodeficiency virus (HIV) co-infection between the two groups (P < 0.05).

**Conclusions:**

HVI in IDU population is a prevalent complication, and is associated with heavy smoking (high number of packs of cigarettes per year), sharing injection equipment, long duration of drug usage, long duration of prison stay, HIV co-infection, history of surgery, blood and blood products transfusion. Older age, longer duration of IDU and imprisonment put the cases at higher risk of acquiring HCV in comparison to HBV.

## Introduction

According to the Iranian Welfare Organization report, out of a government- estimated 1.8 million drug users in Iran, 9 to16 percent are intravenous drug users (IDUs), while half of them shared needles [1]. Thus, it could be estimated that the IDU population in Iran ranges between 200,000 and 300,000. IDUs are increasing in numbers in Iran and constitute an important health problem [2]. Hepatitis C virus (HCV) is widespread among IDUs in parts of Asia. The sharing of injection equipment and imprisonment are the strongest predictors of HCV infection in some areas of Asia, such as Iran [1][3]. The prevalence of HCV infection among IDUs varies in different areas according to socioeconomic and geographical situation as well as HIV infection rate [3][4]. The transmission of hepatitis B virus(HBV), like HCV, is possible in such circumstances as transfusion of unscreened blood and blood products, IDU, vertically from mother to child, because of needle stick, ear piercing, tattoos, barber razors, etc. [5]. The potential outcome of both acute HCV and acute HBV infection leads to persistent chronic infection, cirrhosis, hepatocellular carcinoma and fulminant hepatitis [6]. IDUs all over the world not only have the highest prevalence of HCV and HBV infection but also constitute a potential reservoir of these viruses in the community [7][8]. Published reports have described the viral hepatitis as the most common infectious diseases among this population [9][8][9][10][11][12][13][14][15][16][17].

The HCV and HBV infection rate in Iranian IDU cases is associated with controversial results [7][8][14][15]. However, as the more common routes of transmission for HCV and HBV, such as intravenous drug abuse and needle sharing in Iran [7][15] suggest, research on viral hepatitis is very important. The present study was performed to determine the prevalence and risk factors of HBV and HCV in admitted IDU individuals in Ahvaz, a city located in southwestern Iran.

## Materials and Methods

The study design was a retrospective file audit.

### Subjects

Three hundred and thirty-three injecting drug users were hospitalized from 2002 to 2006 at Razi Hospital, a teaching hospital affiliated to Jundishapur University of Medical Sciences in Ahvaz, the capital city of Khuzestan province in southwestern Iran. These patients were hospitalized due to complaints suggestive of having infectious diseases such as tuberculosis, soft tissue infection, osteomyelitis, endocarditis etc. Participants included all hospitalized IDUs during this period of time and no calculation was made for the sample size. Individual files having serological viral marker tests such as: antibodies to Hepatitis C virus (anti-HCV), hepatitis B surface antigen (HBsAg) and antibody to hepatitis B core antigen (anti-HBc) were created. Cases that fulfilled the diagnostic criteria for hepatitis virus infection (HVI) were included in the present study. Data about age, gender, residency, imprisonment, history of surgery, blood/blood product transfusion, sharing syringes and needles, clinical findings suggestive of hepatitis (e.g., icter, abdominal pain, anorexia,dark urine, malaise and hepatomegaly), laboratory test results of liver biopsy, HCV-RNA-PCR, HCV genotyping(if present) and liver function tests including aminotransferase; Alanin transferase (ALT),Asparagine transferase (AST) were derived and recorded. An ALT and an AST of more than 40 were defined as abnormal aminotransferase. Individuals having at least one of the abovementioned serological viral tests with or without clinical findings were defined as HVI. HVI cases with abnormal liver function tests and clinical findings were defined as symptomatic of hepatitis. Patients were placed in two groups: the HVI group and (non HVI) the NHVI group, and were compared.

### Statistical calculation

Data were analyzed by multivariate logistic regression to control the impact of confounders. Student’s t- test was used to compare mean values and the chi-square test was used to compare the proportion between the two different groups in SPSS for Windows (version 16; SPSS Inc., USA). Results were regarded as significant when P < 0.05.

## Results

Out of a total of 333 IDU cases, 115 cases (34.5%) with a mean age of 24.8±6.2 (range 20-52) were diagnosed as having HVI. Out of a total of 333 IDU cases, 323 (96.9%) were male. According to the health profile of these IDU cases registered at the Khuzestan Health Center (KHC), more than 65% had a history of imprisonment due to addiction and its related social behavior. Thirty-five percent (documented by KHC), mostly below age 20 (89%), had a history of HBV vaccination. The mean duration of illicit drug use was 4.5±1.6 years for the HVI group and 1.8±0.4 years for the NHVI group (P < 0.0001).The duration of time spent in prison for the HVI and the NHVI group was 6.2 ± 4.3 years and 2.5±1.8 years, respectively (P<0.0001. Eighty-five percent of the HVI group and 45% of the NHVI group shared injection equipment (P < 0.005). One hundred and three patients (30.9%) had HCV and 12 (3.6%) had HBV infection. Sixty- two (60.1%) with HCV and 4 (33.3%) with HBV were symptomatic cases of hepatitis. Five asymptomatic cases in the NHVI group had increased ALT and AST, among them two patients under treatment with anti-tuberculosis drugs. The distribution of patients according to gender, age, residency, imprisonment, duration of intravenous (IV) drug abuse and prison stay, history of surgery, blood/blood product transfusion, smoking, cigarette packs per year, coinfection with HIV, clinical findings and laboratory test results are shown inTable 1.

**Table1:** Demographic characteristics, epidemiological and other related data in studied drug abusers.

Variables		H-group (n=115) N (%)	NH-group (n=218) N (%)	P-value
Mean age (years)		24.8 ± 6.2	26.3 ± 5.7	0.02
Gender	Male	111(96.5)	212(97.2)	0.74
	Female	4(3.5)	6(2.8)	
Residency	Urban	102(88.7)	198(90.8)	0.33
	Rural	13(11.3)	20(9.2)	
Imprisonment		81(70.4)	136(62.4)	0.14
Cigarette smoker Mean pack/year		115(100) 17.7 ± 10.4	215(98.6) 11.4 ± 9.6	0.55 <0.0001
History of surgery		15(13.1)	10(4.6)	0.007
Blood transfusion		20(17.4)	11(5.1)	0.0005
Sharing in IV equipment		98(85.2)	98(44.9)	<0.0001
HIV co-infection		41(35.6)	19(8.7)	<0.0001

There was no significant difference in gender, imprisonment, smoking and residency between the two groups (P > 0.05), but there was a significant difference in age, duration of drug abuse, time spent in prison, the sharing of injection equipment, history of surgery, blood transfusion, packs smoked per year, and HIV co-infection between the two groups (P < 0.05). The final multiple logistic regression model (chi-square model = 13.11; P = 0.0001) identified the following as independent risk factors for HVI: a longer duration of intravenous drug use (odds ratio [OR] 3.1; 95% confidence interval [CI] 1.1-8.7) ; sharing in IV equipment (OR 1.9; 95%CI 1.2-2.7); history of surgery (OR 2.8;95% CI 1.3-5.1); blood transfusion (OR 3.4;95% CI 1.5-6.2); and HIV co-infection (OR 4.1;95% CI 1.6-7.1). Means of age, duration of drug abuse and imprisonment are summarized in Table 2. There was a significant difference in age, duration of prison stay and drug abuse between patients with hepatitis B and patients with hepatitis C (P < 0.05).

**Table2:** Mean of age, duration of drug abuse and imprisonment in patients with viral hepatitis in studied drug abusers.

**Variables**	**Hepatitis B N=12**	**Hepatitis C N=103**	**P-value**
Age(year)	26.1 ± 3.9	21.4 ± 5.2	0.02
Drug abuse (year)	3.2 ± 1.8	5.9 ± 2.1	<0.0001
Imprisonment (year)	3.6 ± 3.2	6.7 ± 4.9	0.03

## Discussion

In the present study, the seroprevalence rate of viral hepatitis among IDUs was 34.5%. In earlier studies this rate varies from 1.3% to75 % [18][19].difference in age, duration of drug abuse, time spent in prison, the sharing of injection equipment, history of surgery, blood transfusion, packs smoked per year, and HIV co-infection between the two groups (P < 0.05). The final multiple logistic regression model (chi-square model = 13.11; P = 0.0001) identified the following as independent risk factors for HVI: a longer duration of intravenous drug use (odds ratio [OR] 3.1; 95% confidence interval [CI] 1.1-8.7) ; sharing in IV equipment (OR 1.9; 95%CI 1.2-2.7); history of surgery (OR 2.8;95% CI 1.3-5.1); blood transfusion (OR 3.4;95% CI 1.5-6.2); and HIV co-infection (OR 4.1;95% CI 1.6-7.1).

Means of age, duration of drug abuse and imprisonment are summarized in  Table 2. There was a significant difference in age, duration of prison stay and drug abuse between patients with hepatitis B and patients with hepatitis C

The prevalence of HVI in the IDU population is influenced by many factors such as: the seroprevalence of the most common viral agents (e.g., HCV and HBV) in the community; HBV vaccination coverage; public health status; socioeconomic status; and the number of IDUs who share injection equipment; access to drugs against viral hepatitis; and HIV co-infection [3].

Hepatitis B prevalence in this study was 3.6%. Merat et al. [18], Farhat et al. [19], Sharif et al. [20] in their work reported a rate of 1.3%-4%, whereas this rate in the Todd et al. [21] work was 6.5%. After employment of the HBV vaccination in the Iranian National Program of Immunization-Expanded Program of Immunization (EPI), since 1993 the prevalence of hepatitis B has decreased throughout the country [18][22]. The presence in this study of older patients with hepatitis B, in comparison with hepatitis C, confirms the efficacy of the high coverage of HBV vaccination in those IDU cases who received this vaccine in childhood and adolescence.

The prevalence rate of hepatitis C in our study was 30.9%. The hepatitis C prevalence rate among IDUs varies from 10.5% to 75% in published studies [20][23]. As mentioned previously, this rate is dependent on many factors which vary in different areas according to different socioeconomic status and health facilities. In this study, hepatitis C was more prevalent than hepatitis B. This finding is consistent with areas with similar health conditions [20]. Indeed, HBV vaccination in childhood, the long duration of injection drug use, and more time spent in prison provides the cases with more exposure to HCV than to HBV.

The present study showed that viral hepatitis in the IDU population is associated with sharing injection equipment, a long duration of drug usage, a long duration of prison stay, HIV co-infection, and a history of surgery, blood and blood products transfusion. These findings are in agreement with some earlier studies and literature [3][5][7][8][13][15][17][23]. We believe that these variables may be the main risk factors for viral hepatitis acquisition in the IDU population in the study region.

In our study, viral hepatitis in IDUs is associated with heavy smoking (high number of packs of cigarettes per year). We believe that smoking is not a route of hepatitis transmission but instead is a correlate of hepatitis infection among this group.
